# Psychosocial Effect of Appearance Changes Associated With Cancer: A Scoping Review

**DOI:** 10.7759/cureus.113254

**Published:** 2026-07-23

**Authors:** Madoka Wakabayashi, Koji Tanaka, Kyoko Nagata

**Affiliations:** 1 Graduate School of Medical Sciences, Division of Health Sciences, Kanazawa University, Kanazawa, JPN; 2 Faculty of Health Sciences, Institute of Medical, Pharmaceutical and Health Sciences, Kanazawa University, Kanazawa, JPN

**Keywords:** cancer, complementary/alternative health practices, health and social care policy, mental health, patient experience

## Abstract

This scoping review aimed to synthesize the psychosocial effects of appearance changes and identify knowledge gaps. Cancer, along with its treatment, often leads to changes in appearance. Understanding the psychosocial effects of these changes is crucial for improving the quality of life of cancer survivors. The scoping review of the peer-reviewed literature was conducted using systematic search of PubMed, CINAHL, and PsycARTICLES databases from 2014 to April 2024. Twenty-six studies addressing appearance changes related to specific cancer types, survivor demographics, treatment stages, or providing a comprehensive overview irrespective of cancer type or attributes were reviewed. Key findings were psychosocial effects based on survivors’ age and cancer type, universal psychosocial effects transcending cancer types and treatment methods, the need for appearance-related care, and the gap between survivors' needs and the care provided. Overall, this review highlights that appearance changes are associated with significant psychosocial challenges across diverse groups of cancer survivors and remain insufficiently addressed in current care. To bridge the gap between the needs of cancer survivors and appearance care provided by healthcare professionals, further appearance-related research is essential. Additionally, research should focus on promoting the socialization of appearance care by fostering collaboration not only within the medical field but also with domains such as the beauty industry.

## Introduction and background

According to the International Agency for Research on Cancer, there were approximately 20 million new cases of cancer worldwide in 2022 [1]. Cancer treatment generally involves surgery, systemic therapy, and radiation therapy [2]. During treatment, the patient’s appearance may change due not only to the disease itself but also because of the treatment [3,4]. Common appearance changes include alopecia, surgical scars, skin and nail changes, weight changes, lymphedema, and facial or body disfigurement, depending on the cancer type and treatment modality [3,4]. The concept of appearance care has gained prominence globally since the establishment of the Look Good Feel Better Foundation (formerly the Personal Care Products Council Foundation) in 1989 [5,6]. The Look Good Feel Better Program has been implemented in several countries [6]. Additionally, organizations such as the American Cancer Society, Cancer Research UK, and the Appearance Support Center of the National Cancer Center Japan have actively promoted initiatives related to appearance care, furthering its global reach [7-9]. In recent years, the field of research has also increasingly focused on appearance care, with studies dedicated to the development and enhancement of care products and programs [10-13]. These are important findings in selecting treatment methods to reduce appearance changes and to improve patient satisfaction [10-13]. Therefore, understanding the psychosocial effects of appearance changes is a vital perspective for improving the quality of life of cancer survivors [4,11].

The term “psychosocial” combines the psychological and the social with the implication that the effects of social processes are sometimes mediated through psychological understanding [14]. Cancer survivors are not only individuals who bear the identity of being cancer survivors but also members of society who hold various roles, live in the community, and engage with others in their daily lives [15]. Because appearance changes can affect not only a person’s psychological health but also their social life [4], the term “psychosocial effects” is used in this review.

Given this context, integrating and synthesizing findings on the psychosocial effects of appearance changes associated with cancer is expected to provide new perspectives for addressing these issues beyond the boundaries of specific cancer types or treatment modalities. By focusing on the psychosocial effects directly resulting from appearance changes caused by cancer, this review aims to identify care perspectives that are applicable across cancer types and treatments.

Our research question was formulated using the PCC framework in accordance with the Preferred Reporting Items for Systematic Reviews and Meta-Analyses extension for Scoping Reviews (PRISMA-ScR) guidelines. The Population (P) consisted of people with cancer, the Concept (C) focused on the psychosocial effects of appearance changes and appearance-related needs, and the Context (C) encompassed appearance changes resulting from cancer and its treatment, including appearance-related care provided in healthcare settings.

The objective of this review is to elucidate the psychosocial effects of appearance changes caused by cancer and its treatment or care in order to synthesize the psychosocial effects of appearance changes as well as identify knowledge gaps.

## Review

Review methods

*Study Design*　

A scoping review is the most appropriate methodology for conveying the breadth and depth of available evidence and describing gaps between evidence and practice [16,17]. This review was conducted in accordance with the framework developed by Arksey and O’Malley [18] and further refined by Levac, Colquhoun, and O’Brien [19], using PRISMA-ScR [20]. Thus, the present review comprises the following five phases. These included identification of the research question, identification of relevant studies, study selection, charting of data, and collation and summarization of results [19].

Literature Search

The literature search was conducted using the PubMed, CINAHL, and PsycARTICLES databases, employing the keywords ‘appearance’ AND ‘cancer’ AND ‘psychosocial’.

Inclusion criteria: All types of articles published between 2014 and April 2024, with the full text available, written in English or Japanese, and involving patients with any type of cancer were included.

Exclusion criteria: Non-original articles; studies unrelated to cancer; studies not focusing on the psychosocial effects of appearance changes; studies examining mainly the impact of specific treatments on appearance; studies focusing primarily on the development of scales or tools; studies not targeting cancer survivors as the main subjects were excluded. 

The titles and abstracts of the identified articles were screened based on the predefined inclusion and exclusion criteria by the first author. Potentially eligible articles were then assessed through full-text review. All authors participated in the review process and discussed the findings to reach a consensus on the interpretation of the results. The Mixed Methods Appraisal Tool (MMAT, 2018 version) [21,22], which is a checklist for appraising and/or describing studies in systematic mixed studies reviews, was used to assess the methodological quality of the included studies. Each included study first underwent the two MMAT screening questions to determine whether it was appropriate for appraisal [22]. Each included study was then assessed using the five criteria corresponding to its respective study design [22]. Five studies had two or more MMAT criteria rated as "Can't tell" or "No". However, these studies contain important findings that provide insight into the psychosocial effects of appearance changes. In addition, MMAT generally discourages the exclusion of studies with low methodological quality [22]. Therefore, no study was excluded from this scoping review after quality appraisal. Each study was evaluated according to the relevant criteria based on its study design. The first author conducted the quality appraisal, and the results were reviewed and discussed among all authors to ensure consistency in interpretation. The first author is a combined master's and PhD course post-graduate student studying psycho-oncology and is a licensed nurse. Data from the selected articles were extracted, including the author names, publication year, study aim, study design, research methods, research target, and a summary of the study findings. These data were summarized and categorized into tables while ensuring compliance with copyright regulations.

Results

Selection of Articles

**Figure 1:**
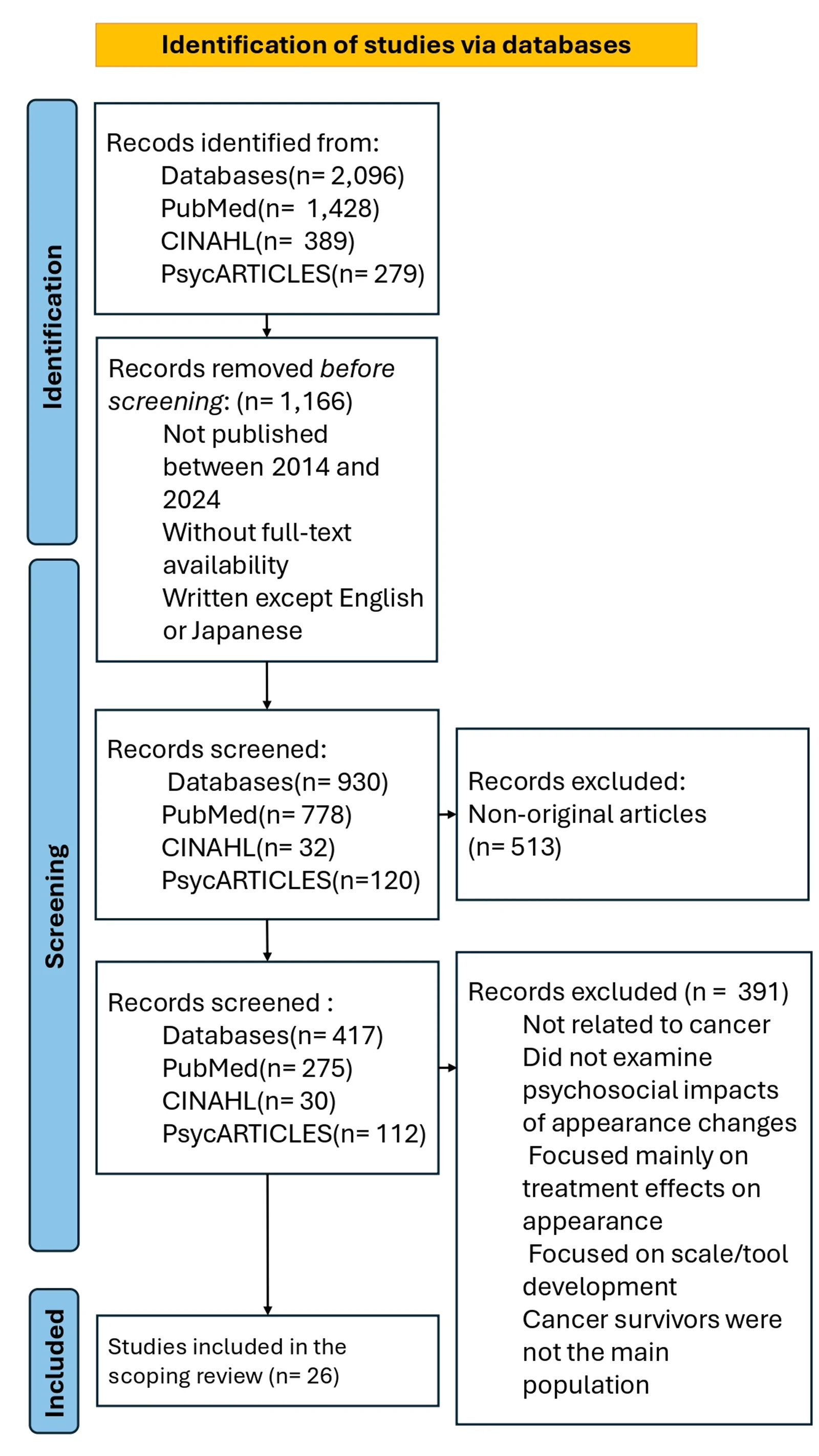
Identification of studies via three databases PRISMA-ScR flow diagram illustrating the study selection process for this scoping review [18]. PRISMA-ScR: Preferred Reporting Items for Systematic Reviews and Meta-Analyses extension for Scoping Reviews

The search results are presented in Figure 1.

A total of 2,096 articles were identified: 1,428 from PubMed, 389 from CINAHL, and 279 from PsycARTICLES. After excluding articles that did not satisfy the inclusion criteria and reviewing the full texts, a total of 26 articles were included in the review. The characteristics of these articles are summarized in Table 1. 

**Table 1:** List of selected articles

No.	Author	Year	Country	Population	Concept	Context	Study design
1	Nikita et al. [23]	2022	India	Head and neck cancer patients	Body image distress	Post-surgical follow-up	Cross-sectional survey
2	Nozawa et al. [24]	2023	Japan	Cancer patients with appearance changes	Distress due to appearance change	Cancer survivorship	Cross-sectional survey
3	Sakai et al. [25]	2020	Japan	Cancer patients undergoing treatment	Psychosocial effects of appearance changes	Clinical oncology setting	Questionnaire survey
4	Brierley et al. [26]	2019	Australia	Adolescent and young adult cancer survivors	Impact of physical appearance change	Survivorship	Qualitative study
5	Crowder et al. [27]	2023	USA	Young adult cancer survivors	Lived experience after cancer treatment	Post-treatment survivorship	Qualitative study
6	Umaretiya et al. [28]	2022	USA	Adolescents and young adults with advanced cancer	Desire for normalcy	Advanced cancer context	Qualitative study
7	Vaarwerk et al. [29]	2019	Netherlands	Survivors of pediatric head-neck rhabdomyosarcoma	Psychosocial well-being	Long-term survivorship	Observational study
8	Cherba et al. [30]	2022	Canada	Head and neck cancer patients	Body image concerns	Surgical consultation	Qualitative study
9	Gibson et al. [31]	2021	Australia	Head and neck cancer survivors	Altered appearance and body image distress	During and after treatment	Qualitative study
10	Ellis et al. [32]	2019	USA	Head and neck cancer patients	Body image disturbance	Post-surgical treatment	Mixed methods
11	McGarvey et al. [33]	2013	Australia	Head and neck cancer patients with lymphoedema	Impact on daily life	Post-treatment survivorship	Qualitative study
12	Reis et al. [34]	2021	Brazil	Head and neck cancer surgery survivors	Appearance concerns and spirituality	Post-surgical survivorship	Cross-sectional study
13	Teo et al. [35]	2016	USA	Head and neck cancer patients	Body image concerns	Reconstruction surgery	Cross-sectional study
14	Levin-Dagan & Baum [36]	2022	Israel	Male breast cancer survivors	Body image after mastectomy	Post-surgical survivorship	Qualitative study
15	Matthews et al. [37]	2018	UK	Breast cancer survivors	Body image and quality of life	Post-mastectomy reconstruction	Qualitative study
16	Zeighami et al. [38]	2018	Iran	Women with breast cancer	Coping with altered body image	Breast cancer survivorship	Qualitative study
17	Patel-Kerai et al. [39]	2016	UK	Breast cancer survivors	Psychosocial experiences	Multi-ethnic survivorship context	Qualitative study
18	Pedersen et al. [40]	2016	Denmark	Women treated for breast cancer	Meaning of weight change and body image	Post-treatment survivorship	Phenomenological study
19	Shammas et al. [41]	2021	USA	Breast reconstruction patients	Dissatisfaction with body image	Post-mastectomy reconstruction	Mixed methods
20	Teo et al. [42]	2016	USA	Breast cancer patients	Body image and quality of life	Breast reconstruction	Cross-sectional study
21	Trusson et al. [43]	2017	UK	Women with breast cancer	Hair loss and cancer identity	Chemotherapy context	Qualitative study
22	D'Hondt et al. [44]	2023	Netherlands	Facial skin cancer patients	Appearance-related psychosocial distress	Post-surgery follow-up	Prospective study
23	Vaidya et al. [45]	2019	USA	Facial skin cancer surgery patients	Appearance-related distress	Dermatologic surgery	Cross-sectional study
24	Tollow et al. [46]	2023	UK	Adults with incurable cancer	Appearance and well-being	Palliative care	Qualitative study
25	Thomas et al. [47]	2014	UK	Cancer survivors with secondary lymphedema	Loss and body image	Chronic survivorship	Qualitative study
26	Charles et al. [48]	2016	France	Cancer patients with targeted therapy	Skin toxicity and body image	Cancer treatment	Prospective study

The MMAT appraisal results for all included studies are presented in Table 2.

**Table 2:** Methodological quality appraisal of the included studies using MMAT (2018) MMAT: Mixed Methods Appraisal Tool

Study	Study design	Criterion 1	Criterion 2	Criterion 3	Criterion 4	Criterion 5
Nikita et al. [23]	Quantitative descriptive study	Yes	Yes	Yes	Yes	Yes
Nozawa et al. [24]	Quantitative descriptive study	Yes	Yes	Yes	Yes	Yes
Sakai et al. [25]	Quantitative descriptive study	Yes	Yes	Yes	Yes	Yes
Brierley et al. [26]	Qualitative study	Yes	Yes	Yes	Yes	Can't tell
Crowder et al. [27]	Qualitative study	Yes	Yes	Yes	Yes	Yes
Umaretiya et al. [28]	Qualitative study	Yes	Yes	Yes	Yes	Yes
Vaarwerk et al. [29]	Quantitative descriptive study	Yes	Yes	Yes	Yes	Yes
Cherba et al. [30]	Qualitative study	Yes	Yes	Yes	Yes	Yes
Gibson et al. [31]	Qualitative study	Yes	Yes	Yes	Yes	Yes
Ellis et al. [32]	Qualitative study	Yes	Yes	Yes	Yes	Yes
McGarvey et al. [33]	Qualitative study	Yes	Yes	Yes	Yes	Can't tell
Reis et al. [34]	Quantitative descriptive study	Yes	Can't tell	Yes	Can't tell	Yes
Teo et al. [35]	Quantitative descriptive study	Yes	Can't tell	Yes	Can't tell	Yes
Levin-Dagan & Baum [36]	Qualitative study	Yes	Yes	Yes	Yes	Yes
Matthews et al. [37]	Qualitative study	Yes	Yes	Yes	Yes	Yes
Zeighami et al. [38]	Qualitative study	Yes	Yes	Yes	Yes	Yes
Patel-Kerai et al. [39]	Quantitative descriptive study	Yes	Yes	Yes	Can't tell	Yes
Pedersen et al. [40]	Qualitative study	Yes	Yes	Yes	Yes	Yes
Shammas et al. [41]	Mixed methods study	Yes	Yes	Yes	Yes	Yes
Teo et al. [42]	Quantitative descriptive study	Yes	Can't tell	Yes	Can't tell	Yes
Trusson et al. [43]	Qualitative study	Yes	Yes	Yes	Yes	Yes
D'Hondt et al. [44]	Quantitative descriptive study	Yes	Yes	Yes	Can't tell	Yes
Vaidya et al. [45]	Quantitative descriptive study	Yes	Can't tell	Yes	Can't tell	Yes
Tollow et al. [46]	Qualitative study	Yes	Yes	Yes	Yes	Yes
Thomas et al. [47]	Qualitative study	Yes	Yes	Yes	Yes	Yes
Charles et al. [48]	Quantitative descriptive study	Yes	Can't tell	Yes	No	Yes

Overview of the Review Findings

The findings of the included studies were synthesized into categories. The key findings are summarized for each category in Table 3.

**Table 3:** Summary of Findings by Category

Category	Key findings	Supporting studies
Psychosocial effects by survivor age	Younger survivors: Greater appearance-related distress and body image concerns. Pediatric survivors: Negative self-evaluation of appearance	[23–29]
Psychosocial effects by cancer type	Head and neck cancer: distress related to visible scars and lymphoedema. Breast cancer: body image disturbance and concerns about femininity and sexuality. Facial skin cancer: persistent appearance-related distress and self-consciousness.	[23,24,29-45]
Universal psychosocial effects beyond cancer types	Identity loss and reduced self-esteem; loss of control over diagnosis disclosure; increased self-consciousness and avoidance of social situations	[23,25,27, 28,31-33, 39,40,43,45–47]
Gaps between survivors’ needs and appearance care	Limited psychosocial support from healthcare professionals; reluctance to discuss appearance concerns in medical settings; need for holistic appearance support throughout the survivorship	[24,25,34,39,46,48]

The findings for each category are described in detail below.

Psychosocial Effects by Survivor Age

Although big differences have been reported among individuals facing distress and difficulty with appearance changes, survivors who are younger and/or female tend to experience higher levels [23-25]. Three studies addressed the adolescent and young adult population [26-28], while one study focused on pediatric patients [29]. For adolescent and young adult survivors, appearance-related changes did not always interfere with daily life, but concerns about appearance or body image were still common [26-28], and appearance changes impacted their psychosocial well-being [26]. In particular, survivors perceived being unrecognized by acquaintances, experiencing a loss of identity, and facing uncontrollable appearance changes as significant events [26]. Not only adolescent and young adult survivors but also pediatric head and neck cancer survivors evaluated their appearance negatively [29]. Across age groups, appearance changes were associated with negative impacts on identity and psychosocial functioning, including body image distress, reduced self-esteem, and social difficulties.

Psychosocial Effects by Cancer Type

A total of 18 studies focused on specific cancer types, including head and neck cancer (eight studies, one involving pediatric cases) [23,29-35], breast cancer (eight studies) [36-43], and skin cancer (two studies, both involving facial skin cancer) [44,45]. Survivors of head and neck cancer often experienced severe distress due to the visibility of scars and lymphoedema in affected areas [31,33]. They reported being concerned about how others perceive their appearance and many of them reported feeling self-conscious [23,32]. Self-consciousness and body image change lead to social avoidance, negatively impacting survivors’ work and their personal life, including romantic relationships [23,32].

Among breast cancer survivors, distress levels and personal assistance requirements were high for patients who experienced mastectomy [24]. Breast cancer is characterized by its location and its effect on sexual well-being [37,41]. The primary motivations for breast reconstruction among survivors were to restore feminine identity and physical attractiveness, safeguard sexual relationships, and maintain a normal appearance in front of their children [38]. Women who elected for reconstruction and who underwent immediate reconstruction had fewer psychosocial adverse effects [37,42]. Many women reported that their feelings of sexual attractiveness returned to preoperative levels after reconstruction [37]. However, their sexual activity did not return, which some women attributed to the influence of their spouse’s reaction to them [37]. Almost all women reported a loss of breast sensitivity following reconstruction, and some women described a sense of sadness about this loss [37]. Male breast cancer survivors described their appearance as “not beautiful” [36]. Men’s negative body image was related not merely to their changes in appearance but also to their being diagnosed with a “woman’s disease,” which undermined their masculine identity [36]. They tried to hide their scars and their breast asymmetry in daily life and felt uncomfortable being in public places where men are typically expected to be topless [36]. When investigating appearance-related changes and psychosocial effects associated with breast cancer, the BREAST-Q, a patient-reported outcome measure to assess the unique outcomes of breast surgery patients, is sometimes used to measure appearance-related distress [41].

Among the studies related to patients with skin cancer, only those focusing on facial skin cancer patients corresponded to the research question of this study. Patients with peripheral lesions or those who underwent secondary intention healing and graft reconstruction reported higher levels of appearance-related psychosocial distress [44]. Although appearance-related psychosocial distress decreases over a longer postoperative period, self-consciousness continued to be reported even in the long-term postoperative phase [45]. When investigating appearance-related changes and psychosocial effects associated with facial skin cancer, the FACE-Q Skin Cancer, an appearance-related psychosocial distress scale, is sometimes used to measure appearance-related distress [44,45]. The BREAST-Q and the FACE-Q Skin Cancer Module are validated, licensed patient-reported outcome measures, and permission for their use must be obtained through the MAPI Research Trust [49,50]. Although these instruments are discussed in this review, no patient-reported outcome measures were directly administered in this study. Among other types of cancer survivors, those who have undergone colostomy surgery in particular experience significant distress and require support [24].

Universal Psychosocial Effects Beyond Cancer Types and Treatments

Survivors are concerned about a wide variety of aspects of their appearance, especially hair, breasts, eyebrows, eyelashes, fingernails, and toenails [25]. Appearance changes can dominate survivors’ sense of identity and affect their perception of themselves as autonomous beings [40,46]. They are described as embodying the diagnosis, experiencing it as a setback [27,46]. Furthermore, they experienced a sense of loss regarding their imagined future and past selves [31]. Cancer-related appearance changes not only have psychological and spiritual impacts on survivors but also exert significant social influence. Appearance changes can cause others to speculate about a patient’s diagnosis or compel patients to disclose their diagnosis to others, stripping them of the right to decide the timing themselves [43,46] and the ability to project an outwardly healthy appearance or lack of visible traces of illness [47]. In relationships with others, changes in appearance lead to altered interactions with the outside world and relationships with others, including increased self-consciousness and avoidance of social situations [23,32,45,46]. Survivors may attempt to conceal their altered appearance not only from others’ eyes but also from their own [47]. This might include avoiding mirrors [31], refusing to be photographed [33], using appearance-care products, and dressing appropriately [31,39]. This is all part of an effort to maintain self-esteem and appear “normal” to others [28]. Incurable cancer adds an additional weight to these experiences and exacerbates feelings of identity loss [46]. However, according to evaluations using the Derriford Appearance Scale, there were no significant differences between survivors with advanced-stage cancer and those with early-stage cancer [25]. This finding suggests that the distress caused by appearance changes is a universal experience that can affect cancer survivors at any stage of the disease.

Gaps Between Survivors’ Needs and Appearance Care

Appearance changes have significant psychosocial effects on cancer survivors. Survivors seek information about appearance-related needs from their doctors before starting treatment, particularly regarding hair loss, edema, and eczema [24,25]. The deterioration of their body image has the potential to adversely impact quality of life and compromise compliance [48]. Satisfaction with one’s physical appearance and having depressive symptoms at the beginning of targeted therapy are important for preventing the deterioration of body image [48]. However, some survivors have reported that healthcare providers adopted attitudes that made them feel that discussing their appearance was inappropriate in the medical setting [46]. Furthermore, even when receiving basic information, discussions about the psychological impact of appearance changes are lacking, highlighting the need for holistic care that includes appearance care [46]. It is suggested that providing care that takes religion into account might maintain or improve appearance-related concerns and body image [34,39]. Moreover, survivors within one year after treatment experienced significant appearance-related concerns, whereas survivors more than three years after treatment reported these concerns resolving over time [27], indicating that patients’ needs for appearance care might change over time.

Discussion

This review found that the psychosocial impacts of changes in appearance due to cancer are influenced by the survivor’s age and cancer type. Younger survivors and women in particular were more susceptible to these impacts. Among cancer types, survivors of head and neck cancer, breast cancer, and skin cancer were found to experience distinct psychosocial effects. This review also identified universal psychosocial impacts of appearance changes that transcend cancer types and survivor demographics. For survivors, changes in appearance were associated with self-consciousness, reduced self-esteem, and loss of identity. These experiences often led to social avoidance and altered interpersonal relationships. Previous reviews of breast cancer survivors have reported that appearance changes are associated with impaired quality of life and loss of identity [51,52], and the findings of the present review are consistent with this evidence. Furthermore, by synthesizing studies across multiple cancer types and survivor populations, this review highlights universal psychosocial impacts of appearance changes that transcend differences in cancer type and survivor characteristics.

The literature contains substantial research on body image, a concept closely related to appearance. Body image refers to subjective experiences related not only to external appearance but also to physical function, ability, and sensations [53]. Although the domains of appearance and body image partially overlap, studies have not consistently distinguished between them. Given that changes in appearance can also alter body image, it is challenging to draw strict boundaries between these two concepts. Studies addressing appearance tend to focus on not only major changes such as alopecia and scarring but also more subtle changes. Thus, it is essential to advance research on appearance and body image while integrating both concepts. This review also identified a gap between the needs of cancer survivors for appearance-related care and the care actually provided. Changes in appearance have a significant psychosocial impact on survivors. However, because healthcare providers are medical professionals, not cosmetic experts, survivors may hesitate to consult with them about appearance-related issues [30,46]. As a result, cancer survivors often seek information from the Web [24] and other sources to address the changes they face and adopt measures such as using appearance care products and adjusting their clothing. However, the information and resources available to survivors are likely influenced by individual opportunities and access. To expand access to appearance care, it is necessary to develop this area beyond the healthcare domain by collaborating with cosmetic and other related fields, establishing it as a form of social care. Previous studies have developed appearance care programs with the involvement of cosmetic professionals, demonstrating their potential to maintain or improve participants’ well-being [11,54,55], and future research should aim to expand and implement these appearance care programs in diverse settings and populations to establish evidence for their broader dissemination and accessibility. Additionally, given the wide range of interests and individual differences in appearance concerns [25], it is crucial to promote the development of appearance care products and broaden the available options for survivors.

This study reviewed the literature on the psychosocial effects of appearance changes as well as studies focusing on body image. This review has several limitations, including cultural and methodological heterogeneity among the included studies and the predominance of qualitative research, which may limit generalizability. Despite these limitations, this review offers a comprehensive mapping of the current evidence and provides a clearer understanding of the key psychosocial dimensions of appearance-related changes among cancer survivors.

## Conclusions

This review identified 26 studies examining the psychosocial impact of appearance changes among cancer survivors. Although the specific experiences varied across cancer types, treatment modalities, and individual characteristics, the findings revealed several universal psychosocial challenges that transcend these differences. Collectively, the evidence underscores that appearance-related distress is not a peripheral issue but a central component of survivorship that significantly influences quality of life. Despite the substantial psychosocial burden associated with appearance changes, a clear gap remains between survivors’ needs and the appearance-related support currently provided by healthcare professionals. The findings highlight the need for healthcare systems to recognize appearance care as an essential aspect of holistic cancer care rather than an optional or cosmetic concern. The synthesis provides important insights into the consistent and pervasive nature of appearance-related distress. Future research should focus on developing and evaluating structured appearance care interventions, establishing clinical guidelines, and promoting the socialization of appearance care beyond medical settings. 
